# A Comparative Study of Glucocorticoids Efficacy in Acute Respiratory Distress Syndrome

**DOI:** 10.3390/ph19010147

**Published:** 2026-01-14

**Authors:** Marian S. Boshra, Mahmoud Ezzat, Mona Ibrahim, Mona Y. Alsheikh, Raghda R. S. Hussein, Marwa Kamal

**Affiliations:** 1 Department of Clinical Pharmacy, Faculty of Pharmacy, Beni-Suef University, Beni-Suef 62511, Egypt; mariansobhy31@yahoo.com (M.S.B.); raghda.hussien@pharm.bsu.edu.eg (R.R.S.H.); 2 Department of Clinical Pharmacy, Faculty of Pharmacy, Fayoum University, Fayoum 63511, Egypt; mam54@fayoum.edu.eg; 3 Department of Chest Diseases, Faculty of Medicine, Fayoum University, Fayoum 63511, Egypt; mia05@fayoum.edu.eg; 4 Department of Pharmacy Practice, Faculty of Pharmacy, King Abdulaziz University, Jeddah 21589, Saudi Arabia; myalsheikh@kau.edu.sa

**Keywords:** ARDS, glucocorticoids, dexamethasone, methylprednisolone, hydrocortisone

## Abstract

**Background:** Acute respiratory distress syndrome (ARDS), recognized as an inflammatory and life-threatening lung injury, is typified by severe hypoxaemia, lack of heart-related pulmonary edema, and bilateral lung infiltrates. Glucocorticoids are anti-inflammatory and immunoregulatory agents that are considered a viable treatment for ARDS. This study sought to contrast the effects of methylprednisolone, hydrocortisone, and dexamethasone at equivalent doses in ARDS. **Methods:** About 195 ARDS patients were allocated at random to take methylprednisolone (1 mg/kg/day), hydrocortisone (350 mg/day), or dexamethasone (13 mg/day). The primary and secondary outcomes over 28 days following the initiation of glucocorticoid therapy involved mortality, ventilator-free days, duration of hospitalization, duration of intensive care unit (ICU), total number of patients requiring invasive mechanical ventilation, and changes in the means of arterial oxygen partial pressure to inspired oxygen fraction (PaO_2_/FiO_2_) and oxygen saturation percentage to inspired oxygen fraction (SpO_2_/FiO_2_) ratios. **Results:** Over the 28-day follow-up, regarding mortality, there was a significant difference between dexamethasone and hydrocortisone, as well as between methylprednisolone and hydrocortisone. However, methylprednisolone exhibited the lowest mortality. There were no significant differences among study groups in ventilator-free days, hospitalization duration, ICU duration, and requirement for invasive mechanical ventilation. On the other hand, methylprednisolone had the lowest means of both durations of hospitalization and ICU, and the lowest requirement for invasive mechanical ventilation. Each study group exhibited a significant increase in both PaO_2_/FiO_2_ and SpO_2_/FiO_2_ ratios at follow-up time. However, dexamethasone showed the highest means of both PaO_2_/FiO_2_ and SpO_2_/FiO_2_ ratios at follow-up time. There was a significant difference in PaO_2_/FiO_2_ and SpO_2_/FiO_2_ ratios at follow-up assessment between dexamethasone and hydrocortisone. **Conclusions:** At equivalent doses, treating ARDS with methylprednisolone may be more successful than using dexamethasone and hydrocortisone.

## 1. Introduction

Acute respiratory distress syndrome (ARDS), identified as an inflammatory, acute, diffuse, and life-threatening lung injury, starts seven days post-triggering event and is marked by lack of heart-related pulmonary edema, bilateral lung infiltrates, and severe hypoxaemia [1]. The predominant cause of ARDS is sepsis. Although pulmonary sepsis, like that resulting from pneumonia, is more widespread, ARDS may result from either non-pulmonary sepsis or pulmonary sepsis from numerous infections. Pancreatitis, several transfusions, gastrointestinal aspiration, and serious trauma linked to shock are the most common non-infectious causes [2].

Developments in clinical and laboratory research have yielded important new understandings of the pathophysiology and pathogenesis of this condition. When the lung is injured by an inflammatory disease, trauma, or infection, inflammatory pathways are activated. Although the inflammatory response helps eradicate pathogens, it can also damage the alveoli, particularly by increasing the permeability of the endothelium and epithelium, which results in the buildup of protein-rich alveolar edema fluid. Because pulmonary edema causes fluid to build up in the lungs’ air passages and interstitium, breathing becomes more difficult, and gas exchange is impaired, leading to hypoxaemia, reduced excretion of carbon dioxide, and ultimately, acute respiratory failure [3]. The ARDS-related morbidity and mortality rates are elevated. About 10% of cases hospitalized in the intensive care unit (ICU) and around 23% of cases using mechanical ventilation are affected. Additionally, the ARDS-related mortality rate varies between 35% and 45% [4].

Prone positioning, hydration management, and lung-protective mechanical ventilation are all part of supportive therapy, which is the usual treatment for severe ARDS. For some cases, veno-venous extracorporeal membrane oxygenation (VV-ECMO) may potentially be necessary, and glucocorticoids and inhaled pulmonary vasodilators can potentially be utilized as adjunctive therapies [5].

**Figure 1:**
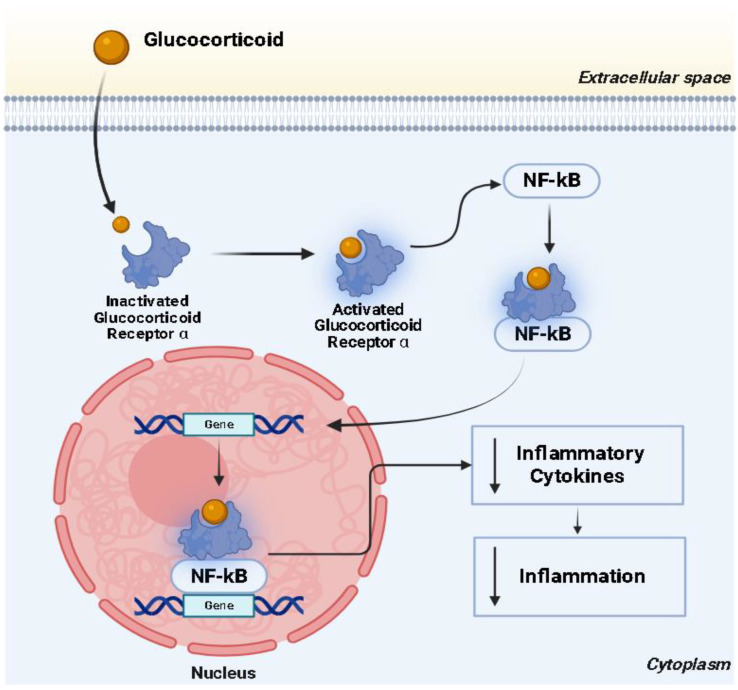
**Mechanism of action of glucocorticoids in ARDS.** This figure illustrates how glucocorticoids work in ARDS. Glucocorticoids bind to the intracellular glucocorticoid receptor α, forming a complex that translocates to the nucleus and inhibits NF-κB signaling. This leads to a decrease in the transcription of pro-inflammatory cytokines, thereby attenuating inflammation. Abbreviations: NF-κB = nuclear factor-kappa B, ↓ = decrease.

Glucocorticoids are anti-inflammatory, antioxidant, anti-fibrotic, and immunoregulatory agents that are thought to be a successful treatment for ARDS [4,6,7,8,9,10,11,12,13,14,15,16,17,18,19]. The clinical consequences remain debatable [20]. The hallmark of ARDS is a sudden respiratory failure resulting from non-cardiovascular pulmonary edema produced by inflammatory cytokines facilitated by nuclear factor-kappa B (NF-κB). Glucocorticoids will probably ameliorate ARDS by reducing NF-κB through the activation of glucocorticoid receptor α, as shown in Figure 1 [21].

In lung tissue, glucocorticoids reduced the expression of pro-inflammatory mediators such as TNF-α, IL-1α, IL-1β, IL-6, and IL-12 p40. They also lessened lung damage by lowering neutrophil-produced oxygen radicals [22]. Additionally, they enhance the expression of IL-1 receptor antagonist and cooperate with natural anti-inflammatory cytokines such as IL-4, IL-10, and IL-13. By inhibiting cytokines, glucocorticoids have an inhibitory action on fibrin pathways, including collagen deposition and fibroblast proliferation. They promote monocyte, T-cell, and eosinophil apoptosis, which may naturally reduce inflammation in ARDS [8].

Glucocorticoids have been utilized in the initial ARDS stage, distinguished by pronounced inflammation, and in the late ARDS stage, marked by increased lung fibrosis. The features of these two stages vary markedly, describing the observed discrepancies in the actions of glucocorticoids under these unique circumstances [22].

The chief target of this research is to contrast the effects of methylprednisolone, hydrocortisone, and dexamethasone at equivalent doses in ARDS.

## 2. Results

**Figure 2:**
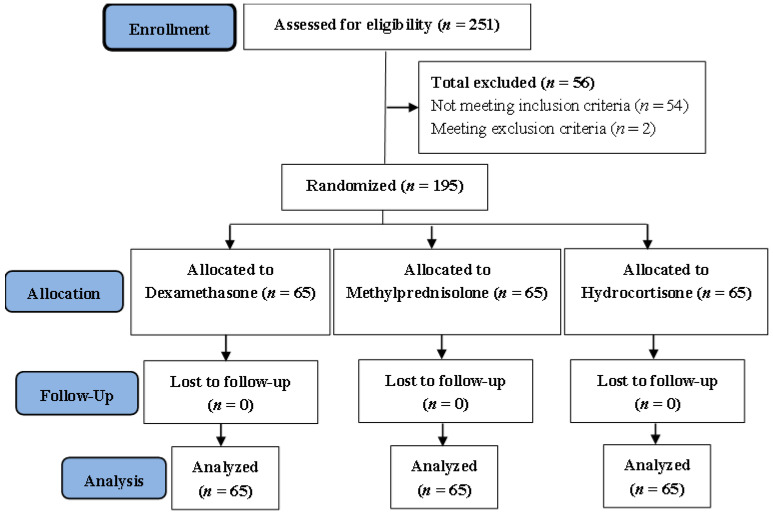
**Trial Flow Diagram.** This diagram illustrates the flow of patients through each stage of the study: enrollment, randomization, allocation, follow-up, and analysis.

According to the trial flow diagram (Figure 2), of the 251 patients whose eligibility was evaluated, around 56 were excluded based on the criteria for inclusion and exclusion between 1 August 2024 and 1 April 2025. Finally, 195 patients satisfying the criteria for inclusion were split up into three groups at random: (1) Group 1: 65 patients received dexamethasone; (2) Group 2: 65 patients received methylprednisolone; (3) Group 3: 65 patients received hydrocortisone. No patients were lost to follow-up within every group. All patients in each group were analyzed.

### 2.1. Baseline Clinical and Demographic Information

In Table 1, Table 2, Table 3 and Table 4, the baseline clinical and demographic information for every study group is summarized. There were no significant differences between the three glucocorticoid treatments concerning age, height, body weight, sex distribution, vital signs, comorbidities, smoking status, nutrition history, causes and severity of ARDS, time from symptom onset to glucocorticoid therapy, days on glucocorticoid therapy, and medications and treatments.

### 2.2. Primary and Secondary Outcomes

**Figure 3:**
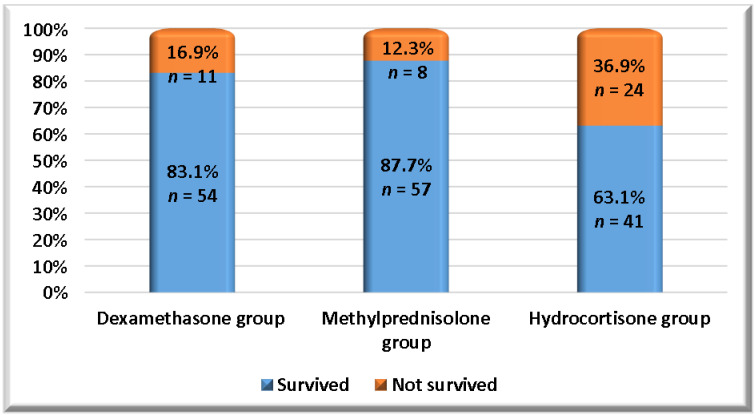
**Mortality throughout 28 days in study groups.** This figure illustrates the percentage of survivors and non-survivors in each study group.

As shown in Table 5 and Figure 3, a summary of each study group’s primary and secondary outcomes is provided. In terms of mortality, a significant difference was observed between dexamethasone and hydrocortisone, as well as between methylprednisolone and hydrocortisone. However, methylprednisolone exhibited the lowest mortality. Regarding ventilator-free days, hospitalization duration, ICU duration, and the requirement for invasive mechanical ventilation, no significant differences were observed between study groups in these variables. On the other hand, methylprednisolone had the lowest means of both durations of hospitalization and ICU, and the lowest requirement for invasive mechanical ventilation.

### 2.3. Comparisons of PaO_2_/FiO_2_ and SpO_2_/FiO_2_ Ratios in Different Study Groups

**Figure 4:**
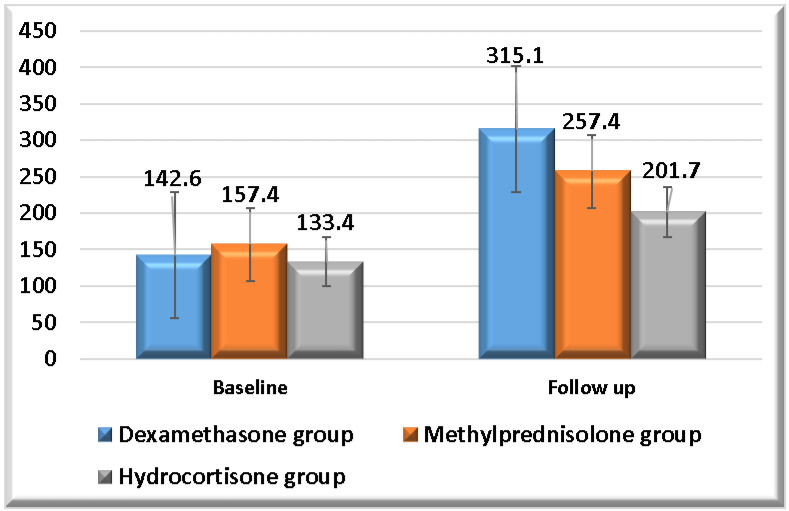
**PaO_2_/FiO_2_ ratio follow-up in study groups.** This figure illustrates the means of the PaO_2_/FiO_2_ ratio at baseline and follow-up in each study group.

A summary of the comparisons of the ratios of arterial oxygen partial pressure to inspired oxygen fraction (PaO_2_/FiO_2_) and oxygen saturation percentage to inspired oxygen fraction (SpO_2_/FiO_2_) for each study group is provided in Table 6 and Figure 4 andFigure 5. Each study group exhibited a significant increase in both PaO_2_/FiO_2_ and SpO_2_/FiO_2_ ratios at follow-up time. On the other hand, dexamethasone showed the highest means of both PaO_2_/FiO_2_ and SpO_2_/FiO_2_ ratios at follow-up time. There were no significant differences in PaO_2_/FiO_2_ and SpO_2_/FiO_2_ ratios at baseline assessment between the three study groups. There were no significant differences in PaO_2_/FiO_2_ and SpO_2_/FiO_2_ ratios at follow-up assessment between dexamethasone and methylprednisolone, as well as between methylprednisolone and hydrocortisone. However, there was a significant difference in PaO_2_/FiO_2_ and SpO_2_/FiO_2_ ratios at follow-up assessment between dexamethasone and hydrocortisone.

### 2.4. Adverse Effects

No adverse effects caused by glucocorticoids were observed in any of the patients in each study group throughout the study.

## 3. Discussion

This research sought to contrast the effects of methylprednisolone, hydrocortisone, and dexamethasone at equivalent doses in ARDS. Throughout the 28-day follow-up, there was a significant difference between dexamethasone and hydrocortisone, as well as between methylprednisolone and hydrocortisone, in mortality. However, methylprednisolone exhibited the lowest mortality. There were no significant differences between study groups in ventilator-free days, hospitalization duration, ICU duration, and the requirement for invasive mechanical ventilation. On the other hand, methylprednisolone had the lowest means of both durations of hospitalization and ICU, and the lowest requirement for invasive mechanical ventilation. Each study group exhibited a significant increase in both PaO_2_/FiO_2_ and SpO_2_/FiO_2_ ratios at follow-up time. However, dexamethasone showed the highest means of both PaO_2_/FiO_2_ and SpO_2_/FiO_2_ ratios at follow-up time. There were no significant differences in PaO_2_/FiO_2_ and SpO_2_/FiO_2_ ratios at baseline assessment between the three study groups. There was a significant difference in PaO_2_/FiO_2_ and SpO_2_/FiO_2_ ratios at follow-up assessment between dexamethasone and hydrocortisone.

Research on glucocorticoid effectiveness in ARDS has been contradictory. The effectiveness of glucocorticoids in ARDS is supported by various studies; Zayed et al.’s meta-analysis, which included 8 studies with 1091 patients, indicated that glucocorticoid administration in ARDS reduced mortality and increased ventilator-free days [4]. Lin et al. proved in a meta-analysis involving 9 studies including 1371 patients that glucocorticoids might increase ventilator-free days, reduce mortality, and enhance PaO_2_/FiO_2_ ratio in ARDS [6]. In a meta-analysis involving 18 meta-analyses, with 38 primary studies that included 3760 patients, Rashid et al. revealed that glucocorticoids could increase ventilator-free days and decrease ICU duration in ARDS. In addition, it could non-significantly reduce mortality [7]. In a systematic review encompassing 15 studies with 8877 patients, Landolf et al. demonstrated that glucocorticoids ameliorated mortality and ventilator-free days in coronavirus disease 2019 (COVID-19) and non-COVID-19 ARDS [8]. In a multicenter retrospective study performed on 508 ARDS patients, Zhang et al. explained that glucocorticoids could lower ICU mortality in ARDS [9].

Glucocorticoid dose, duration, and initiation timing are critical factors in the management of ARDS and are controversial among studies; Zhao et al., in a meta-analysis involving 10 studies, revealed that when started early, low-dose glucocorticoid treatment over a long time decreased mortality linked to ARDS [10]. Sun et al. conducted a meta-analysis including 12 studies involving 1505 ARDS patients, demonstrating that low-dose and early glucocorticoid therapy increased ventilator-free days, decreased mortality, and improved the PaO_2_/FiO_2_ ratio in ARDS [11]. In an umbrella review involving 10 meta-analyses and systematic reviews with 8491 patients, Abate et al. showed that early low-dose glucocorticoids decreased mortality and raised ventilator-free days in ARDS [12]. Mammen et al., in a meta-analysis involving 7 studies with 851 patients, explained that early systemic glucocorticoids in ARDS might decrease mortality and increase ventilator-free days [13].

Several studies prove the efficacy of methylprednisolone in ARDS. In a case series involving 7 patients with COVID-19 ARDS, So et al. explained that short-term, high-dose early methylprednisolone treatment (within 13 days, a 3-day 1000 or 500 mg/day intravenous methylprednisolone, then 1 mg/kg/day, and subsequently tapered by 10 or 20 or 30 mg/day oral prednisolone, ending at 10 mg) might offer a favorable prognosis for COVID-19 ARDS patients. In addition, when methylprednisolone was started, a 100% survival rate and weaning from mechanical ventilation occurred in all patients within seven days [14]. In a meta-analysis involving 7 studies with 996 patients, Li et al. explained that low- and medium-dose methylprednisolone could lower mortality and raise ventilator-free days for 28 days in ARDS [15]. Boglione et al., in a single-center, retrospective analysis involving 83 COVID-19 ARDS patients, demonstrated that early methylprednisolone at high dose (5–8 mg/kg/day) for 2 days lowered mortality and hospitalization period in COVID-19 ARDS patients [16].

Several studies prove the effectiveness of dexamethasone in ARDS; in a multicenter, randomized controlled study comprising 277 patients, Villar et al. showed that in moderate to severe ARDS, early intravenous dexamethasone (20 mg/day for 5 days, which was reduced to 10 mg/day for another 5 days) could decrease mortality and increase ventilator-free days [17]. In a multicenter, randomized trial carried out in 41 ICUs in Brazil involving 299 COVID-19 ARDS patients, Tomazini et al. proved that intravenous dexamethasone (20 mg/day for 5 days, then 10 mg/day for another 5 days) statistically significantly increased ventilator-free days throughout 28 days [18].

Several studies prove the effect of hydrocortisone in ARDS; Tongyoo et al., in a trial with 197 patients, proved that early intravenous hydrocortisone (50 mg) every 6 h for 7 days improved lung function in sepsis-associated ARDS [19].

Numerous studies dispute the effects of glucocorticoids in ARDS; in a meta-analysis, included 14 studies with 1362 patients, Junhai et al. found that glucocorticoids had no discernible impact on mortality in ARDS [23]. In a study with 1831 patients with ARDS, Lu et al. demonstrated that glucocorticoid therapy lowered ventilator-free days and lengthened ICU stay. In addition, it did not affect 28-day mortality [24]. In a trial involving 50 COVID-19 ARDS patients, Jamaati et al. demonstrated that glucocorticoid administration (intravenous dexamethasone at a dose of 20 mg/day for 5 days, then at 10 mg/day for another 5 days) had no benefit in COVID-19 ARDS [25]. Tsai et al. revealed in a nationwide multicenter study that included 241 influenza-related ARDS patients who received invasive mechanical ventilation that early glucocorticoid therapy (hydrocortisone at an equivalent dose of ≥200 mg throughout three days) was linked to a noticeably increased mortality at the hospital among adult patients suffering from influenza-related ARDS [26]. In a meta-analysis encompassing 17 studies with 2508 ARDS patients, Wu et al. proved that glucocorticoid therapy did not lower 28-day mortality [27]. Yoshihiro et al. proved in a network meta-analysis involving 9 studies with 1212 ARDS patients that dexamethasone, hydrocortisone, low-dose methylprednisolone, and high-dose methylprednisolone did not lower mortality [21].

Numerous studies show that methylprednisolone is more effective than hydrocortisone and dexamethasone; in a study involving 414 mechanically ventilated COVID-19 ARDS patients, Saeed et al. demonstrated that intravenous methylprednisolone (2 mg/kg/day), in contrast to intravenous dexamethasone (6 mg/day), improved ICU duration, the mortality, and increased ventilation-free days when given for 10 days [28]. These results resemble the findings of this study, although the larger sample size and different doses and durations of glucocorticoids used in their research.

In a study involving 85 COVID-19 ARDS patients, Abdelkader et al. demonstrated that intravenous methylprednisolone (1 mg/kg/day) decreased mortality, shortened hospitalization duration, and decreased need for ventilation in COVID-19 ARDS, in contrast to intravenous dexamethasone (6 mg/day) [29]. Their study agrees with this study’s findings, despite of small sample size, inclusion of only COVID-19 ARDS patients, and dexamethasone different dose.

Elbrassi et al., in a study, demonstrated that methylprednisolone (as a loading dose of 1 mg/kg subsequently by an infusion of 1 mg/kg/day for 7 days, then gradual tapering over 14 days), when compared to hydrocortisone (50 mg every six hours for one week), showed more benefit in the reduction in mortality at the hospital and the improvement of PaO_2_/FiO_2_ ratio in ARDS. However, there were no significant differences regarding ICU duration and decreased ventilation duration [30]. Their results align with this study despite differences in hydrocortisone dose and glucocorticoid therapy duration.

In a meta-analysis encompassing 17 studies with 8592 patients, Jiang et al. demonstrated that methylprednisolone could lower mortality in COVID-19 ARDS, but other glucocorticoids did not. However, all glucocorticoids might elevate ventilator-free days [31]. Although studies involving COVID-19 ARDS patients have been included, their results agree with this study.

In a meta-analysis encompassing 14 studies with 1607 patients, Chang et al. demonstrated that hydrocortisone and methylprednisolone might lower the mortality of ARDS patients, with methylprednisolone showing the best results. Dexamethasone, however, had no discernible effect [32]. These results are concordant with this study except for dexamethasone, which exhibited lower mortality compared to hydrocortisone in this study.

Some studies disagree with this study’s findings; in a prospective randomized study including 106 COVID-19 ARDS patients, Taher et al. demonstrated that for up to 10 days, dexamethasone (6 mg/day), when compared to methylprednisolone (16 mg two times a day) or hydrocortisone (50 mg three times a day), showed a more favorable impact on requirement for invasive mechanical ventilation, mortality, PaO_2_/FiO_2_ ratio, and ICU and hospitalization duration [33]. Their study’s small sample size, inclusion of only COVID-19 ARDS patients, and different glucocorticoid therapy durations and dosages may be the cause of this contradictory finding. However, their study agrees with this study in that dexamethasone improved the PaO_2_/FiO_2_ ratio compared to methylprednisolone and hydrocortisone.

The limitations of this study were as follows: (1) it was conducted at a single center; the findings may not be representative of others; (2) it did not include a control group (patients not receiving any glucocorticoids); (3) it did not take into consideration the immunologic status of patients.

## 4. Materials and Methods

### 4.1. Study Design

This prospective, randomized, three-arm, double-blind, controlled study was conducted at the Respiratory ICU of the Department of Chest Diseases at Fayoum University from 1 August 2024 to 1 April 2025. In this study, 195 inpatients who fulfilled the inclusion criteria were enrolled after providing written informed consent. Approximately 195 ARDS patients were split up into three groups in a 1:1:1 ratio. There were 65 ARDS patients in each group. The first patient was enrolled on 1 August 2024, and the assessment of the last patient was completed on 1 April 2025.

### 4.2. Ethical Considerations

The Ethics Committee of the Faculty of Medicine at Fayoum University approved this study under approval number (M 727) in its session (119) on 9 June 2024. Before enrollment in the study, the patients provided written informed consent. Registration of this study was done on ClinicalTrials.gov under registration number (NCT06496997), available from (https://clinicaltrials.gov/study/NCT06496997, accessed on 4 July 2024).

### 4.3. Inclusion Criteria

This research comprised mechanically ventilated patients who fulfilled the Berlin definition of ARDS [34] as follows: (1) acute hypoxemia, identified as PaO_2_/FiO_2_ ratio of less than 300 mm Hg; (2) emergence or exacerbation of pulmonary symptoms within 7 days post-insult; (3) bilateral opacities are not entirely defined by nodules, effusions, or lobar or lung collapse on computed tomography (CT) or chest x-ray; (4) heart failure is not the main reason for acute respiratory failure.

### 4.4. Exclusion Criteria

Patients who experienced acute respiratory failure as a result of heart failure were excluded.

### 4.5. Intervention

Qualified patients were split up into three groups at random in a 1:1:1 ratio: group 1 took intravenous dexamethasone at an equivalent dosage of 13 mg/day; group 2 took intravenous methylprednisolone at an equivalent dosage of 1 mg/kg/day (averaging 70 mg/day based on 70 kg body weight); and group 3 took intravenous hydrocortisone at an equivalent dosage of 350 mg/day.

The doses of the three glucocorticoids were calculated depending on the low dose of methylprednisolone (1 mg/kg/day), thought to be used in ARDS, based on the ARDS Clinical Practice Guideline 2021 [35]. The average body weight of 70 kg was used to calculate the dose of methylprednisolone (1 mg/kg/day * 70 kg = 70 mg/day), and the methylprednisolone dose of 70 mg/day was used to calculate the equivalent doses of both dexamethasone (13 mg/day) and hydrocortisone (350 mg/day) using MDCalc website (https://www.mdcalc.com/, accessed on 6 May 2024).

The Patients took the study drugs throughout their hospital stay. All of the study drugs were stopped without tapering off at the patients’ hospitalization conclusion.

Non-invasive mechanical ventilation involved alternating between continuous positive airway pressure (CPAP) and bilevel positive airway pressure (BiPAP). For invasive mechanical ventilation, volume-controlled ventilation (VCV) mode was used.

Lung protective ventilation was used with tidal volume (4–8 mL/kg), plateau pressure less than 30 cm H_2_O, and high positive end-expiratory pressure (PEEP) values without recruitment maneuvers based on the American Thoracic Society (ATS) guidelines [36].

Prone positioning, recruitment maneuvers, inhaled pulmonary vasodilators, neuromuscular blocking agents, and VV-ECMO were not used in any of the patients.

### 4.6. Randomization

For randomization, block randomization was employed with a block size of 6 in a 1:1:1 ratio. The randomization was supplied by an impartial statistician.

### 4.7. Blinding

This study was double-blind. Neither the study participants nor the researchers were aware of the treatment allocation. The unblinded nurse staff prepared the study medications.

### 4.8. Data Collection

Data was gathered from the patients’ records. Microsoft Excel was used to establish an electronic database into which the collected data was input following the treating physician’s review and approval. Clinical outcomes, the number of days receiving glucocorticoid therapy, underlying diseases, demographic data, in-hospital treatments, radiologic and laboratory findings when enrolling, and clinical signs and symptoms when enrolling were all collected. Every patient was followed up from the day of enrollment throughout 28 days after their allocation at random.

### 4.9. Study Outcomes

The primary outcome was the mortality throughout 28 days following enrollment and the secondary outcomes throughout 28 days following enrollment were as follows: (1) total number of patients requiring invasive mechanical ventilation; (2) ventilator-free days; (3) duration of ICU; (4) duration of hospitalization; (5) changes in the means of PaO_2_/FiO_2_ and SpO_2_/FiO_2_ ratios over 28 days following the initiation of glucocorticoid therapy.

### 4.10. Sample Size

G-Power© software, version 3.1.7 (Institute of experimental psychology, Heinrich Heine University, Düsseldorf, Germany), was employed to determine the sample size. Each group had a minimum sample size of 65 patients. The sample size was determined using prior study findings [19]. The power was 80%, the effect size was 0.441, and the two-sided (two-tailed) type I error was 0.05.

### 4.11. Statistical Analysis

Analysis of the data was done using the Statistical Package of Social Science (SPSS) software, version 22 (SPSS Inc., Chicago, IL, USA). For simple descriptive analysis, Numerical and percentage representations were utilized for qualitative data, and means and standard deviations were utilized for quantitative parametric data, and medians and ranges were utilized for quantitative non-parametric data. For quantitative parametric data, the independent samples *t*-test, the one-way analysis of variance (ANOVA) test, with the Bonferroni post hoc test, and the paired *t*-test were utilized. For quantitative non-parametric data, the Kruskal–Wallis test and the Friedman test were utilized. For qualitative data, the chi-square test and the McNemar test were utilized. A *p*-value less than 0.05 was deemed statistically significant.

### 4.12. Safety Assessment

The assessment of glucocorticoids’ adverse effects was done by the nursing staff through patients’ records, signs and symptoms, and laboratory testing.

## 5. Conclusions

At equivalent doses, treating ARDS with methylprednisolone may be more successful than using dexamethasone and hydrocortisone. Methylprednisolone reduced mortality, hospitalization duration, ICU duration, and the requirement for invasive mechanical ventilation compared to the other two drugs. The limitations of the current study include being a single-center study. Hence, larger multicenter studies need to be conducted in the future.

## Figures and Tables

**Figure 5:**
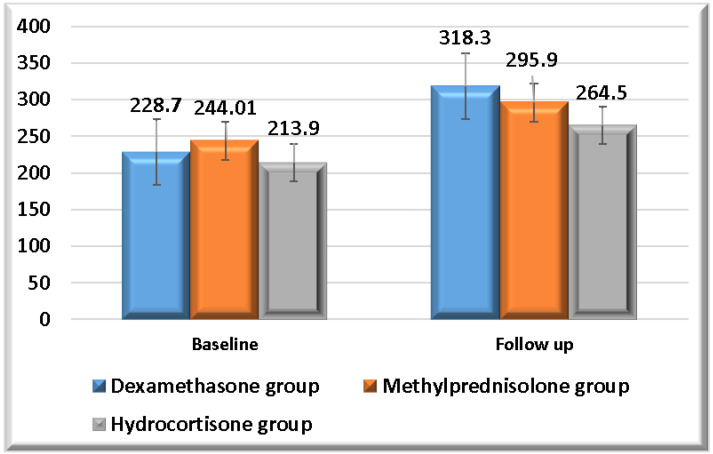
**SpO_2_/FiO_2_ ratio follow-up in study groups.** This figure illustrates the means of the SpO_2_/FiO_2_ ratio at baseline and follow-up in each study group.

**Table 1:** **Comparisons of age, sex, baseline vital signs, and smoking status in different study groups.** Variables compared include age; sex (male or female); vital signs (temperature, systolic and diastolic blood pressures, and heart and respiratory rates); and smoking status (non-, active, or ex-smoker).

Variables	Dexamethasone Group (*n* = 65)		Methylprednisolone Group (*n* = 65)		Hydrocortisone Group (*n* = 65)		*p*-Value
	Mean	SD	Mean	SD	Mean	SD	
Age (years)	61.9	13.7	60.9	15.6	65.9	13.9	0.12 @
**Sex**	**No.**	**(%)**	**No.**	**(%)**	**No.**	**(%)**	
Male	37	56.9%	30	46.2%	34	52.3%	0.47 $
Female	28	43.1%	35	53.8%	31	47.7%	
**Baseline vital signs**	**Mean**	**SD**	**Mean**	**SD**	**Mean**	**SD**	
Temperature (°C)	37.2	0.48	37.2	0.38	37.2	0.31	0.91 @
Systolic blood pressure (mmHg)	123.4	20.5	120.2	24.9	114.5	23.2	0.08 @
Diastolic blood pressure (mmHg)	76.5	11.5	74.6	13.9	71.5	13.6	0.09 @
Heart rate (bpm)	99	18	96	17	95	18	0.22 @
Respiratory rate (bpm)	27	18	26	13	22	6	0.12 @
**Smoking status**	**No.**	**(%)**	**No.**	**(%)**	**No.**	**(%)**	
Nonsmoker	45	69.2%	42	64.6%	49	75.4%	0.75 $
Active smoker	16	24.6%	18	27.7%	12	18.5%	
Ex-smoker	4	6.2%	5	7.7%	4	6.2%	

@: ANOVA test, $: Chi-square test.

**Table 2:** **Comparisons of comorbidities in different study groups.** Variables compared include diabetes, hypertension, cardiovascular disease, respiratory disease, COPD, interstitial lung disease, pleural effusion, neurological disease, kidney disease, liver disease, cancer, and other comorbidities.

Variables	Dexamethasone Group (*n* = 65)		Methylprednisolone Group (*n* = 65)		Hydrocortisone Group (*n* = 65)		*p*-Value
	No.	(%)	No.	(%)	No.	(%)	
**Comorbidities**							
Diabetes	15	23.1%	17	26.2%	13	20%	0.71 $
Hypertension	22	33.8%	28	43.1%	25	38.5%	0.55 $
Cardiovascular disease	6	9.2%	10	15.4%	10	15.4%	0.49 $
Respiratory disease	65	100%	65	100%	65	100%	----
Chronic obstructive pulmonary disease (COPD)	33	50.8%	28	43.1%	22	33.8%	0.15 $
Interstitial lung disease	6	9.2%	8	12.3%	6	9.2%	0.80 $
Pleural effusion	8	12.3%	9	13.8%	15	23.1%	0.20 $
Neurological disease	3	4.6%	2	3.1%	3	4.6%	0.87 $
Kidney disease	2	3.1%	3	4.6%	6	9.2%	0.29 $
Liver disease	0	0%	2	3.1%	4	6.2%	0.13 $
Cancer	8	12.3%	8	12.3%	13	20%	0.36 $
Other comorbidities	4	6.2%	8	12.3%	8	12.3%	0.41 $

$: Chi-square test.

**Table 3:** **Comparisons of severity and causes of ARDS in different study groups.** Variables compared include ARDS severity (mild, moderate, or severe) and ARDS causes (sepsis, pneumonia, aspiration pneumonia, pulmonary contusion, and pulmonary embolism).

Variables	Dexamethasone Group (*n* = 65)		Methylprednisolone Group (*n* = 65)		Hydrocortisone Group (*n* = 65)		*p*-Value
	No.	(%)	No.	(%)	No.	(%)	
**Severity of ARDS**							
Mild	14	21.5%	15	23.1%	13	20%	0.18 $
Moderate	30	46.2%	36	55.4%	25	38.5%	
Severe	21	32.3%	14	21.5%	27	41.5%	
**Causes of ARDS**							
Sepsis	35	53.8%	35	53.8%	32	49.2%	0.94 $
Pneumonia	24	36.9%	25	38.5%	24	36.9%	
Aspiration Pneumonia	2	3.1%	3	4.6%	3	4.6%	
Pulmonary Contusion	2	3.1%	1	1.5%	4	6.2%	
Pulmonary embolism	2	3.1%	1	1.5%	2	3.1%	

$: Chi-square test.

**Table 4:** **Comparisons of medications and treatments during hospitalization in different study groups.** Variables compared include time from symptom onset to glucocorticoid therapy, days on glucocorticoid therapy, antibiotics, anticoagulants, proton pump inhibitors, antipyretics, sedatives, diuretics, and dialysis.

Variables	Dexamethasone Group (*n* = 65)		Methylprednisolone Group (*n* = 65)		Hydrocortisone Group (*n* = 65)		*p*-Value
	Mean ± SD	Median (Range)	Mean ± SD	Median (Range)	Mean ± SD	Median (Range)	
Time from symptom onset to glucocorticoid therapy (days)	6.5 ± 6.2	5 (1–30)	7.5 ± 10.3	4 (1–60)	5.5 ± 7.2	5 (1–30)	0.39 Ω
Days on glucocorticoid therapy (days)	7.6 ± 4.6	7 (2–25)	7.1 ± 1.7	7 (1–10)	7.3 ± 5.2	7 (1–22)	0.99 Ω
**Antibiotics**	**No.**	**(%)**	**No.**	**(%)**	**No.**	**(%)**	
According to the Community-acquired pneumonia (CAP) guidelines	16	24.6%	10	15.4%	9	13.8%	0.22 $
According to the Hospital-acquired pneumonia (HAP) guidelines	49	75.4%	55	84.6%	56	86.2%	
**Anticoagulants**	**No.**	**(%)**	**No.**	**(%)**	**No.**	**(%)**	
No	8	12.3%	6	9.2%	10	15.4%	0.53 $
Enoxaparin	56	86.2%	59	90.8%	55	84.6%	
Fondaparinux	1	1.5%	0	0%	0	0%	
**Other treatments**	**No.**	**(%)**	**No.**	**(%)**	**No.**	**(%)**	
Proton pump inhibitors (PPIs) (Pantoprazole)	53	81.5%	57	87.7%	60	92.3%	0.18 $
Antipyretics (Acetaminophen)	15	23.1%	12	18.5%	22	33.8%	0.12 $
Sedatives (Midazolam)	19	29.2%	16	24.6%	25	38.5%	0.22 $
Diuretics (Furosemide)	12	18.5%	14	21.5%	19	29.2%	0.32 $
Dialysis	1	1.5%	1	1.5%	3	4.6%	0.44 $

Ω: Kruskal–Wallis test, $: Chi-square test.

**Table 5:** **Primary and secondary outcomes throughout 28 days in different study groups.** Outcomes compared include mortality, requirement for invasive mechanical ventilation, hospitalization duration, ICU duration, and ventilator-free days.

Variables	Dexamethasone Group (*n* = 65)		Methylprednisolone Group (*n* = 65)		Hydrocortisone Group (*n* = 65)		*p*-Value
	Mean	SD	Mean	SD	Mean	SD	
Duration of ICU stay (days)	8.5	6.01	6.9	3.9	8.6	5.2	0.13 Ω
Duration of hospital stay (days)	8.6	5.9	7.4	4.1	9.4	6.1	0.11 Ω
Ventilator-free days	6.3	4.3	6.2	3.5	6.9	5.3	0.56 Ω
**Requirement for invasive mechanical ventilation**	**No.**	**(%)**	**No.**	**(%)**	**No.**	**(%)**	
No	46	70.8%	50	76.9%	40	61.5%	0.22 $
Yes	19	29.2%	15	23.1%	25	38.5%	
**Mortality**	**No.**	**(%)**	**No.**	**(%)**	**No.**	**(%)**	
Survived	54	83.1%	57	87.7%	41	63.1%	0.45 $ 0.01 *b $ 0.001 *c $
Not survived	11	16.9%	8	12.3%	24	36.9%	

Ω: Kruskal–Wallis test, $: Chi-square test. *b: significant difference between dexamethasone and hydrocortisone. *c: significant difference between methylprednisolone and hydrocortisone.

**Table 6:** **Comparisons of PaO_2_/FiO_2_ and SpO_2_/FiO_2_ ratios in different study groups.** PaO_2_/FiO_2_ and SpO_2_/FiO_2_ ratios are compared at baseline and follow-up.

Variables	Dexamethasone Group (*n* = 65)		Methylprednisolone Group (*n* = 65)		Hydrocortisone Group (*n* = 65)		*p*-Value
	Mean	SD	Mean	SD	Mean	SD	
**PaO_2_/FiO_2_ ratio**							
Baseline	142.6	62.8	157.4	91.8	133.4	65.1	0.09 €
Follow up	315.1	225.2	257.4	200.2	201.7	100.1	0.22 € 0.002 *b € 0.26 €
***p*-value**	**<0.001 # ¥**		**<0.001 # ¥**		**<0.001 # ¥**		
**SpO_2_/FiO_2_ ratio**							
Baseline	228.7	99.4	244.01	91.7	213.9	97.7	0.21 €
Follow up	318.3	109.1	295.9	107.7	264.5	110.04	0.73 € 0.01 *b € 0.30 €
***p*** **-value**	**<0.001 # ¥**		**0.001 # ¥**		**0.008 # ¥**		

€: Friedman test, ¥: Paired *t*-test. *b: significant difference between dexamethasone and hydrocortisone. # significance in follow-up measures in each group.

## Data Availability

The original contributions presented in the study are included in the article; further inquiries can be directed to the corresponding author.

## Abbreviations

The following abbreviations are used in this manuscript:

ANOVA	Analysis of variance
ARDS	Acute respiratory distress syndrome
ATS	American Thoracic Society
BiPAP	Bilevel positive airway pressure
COVID-19	Coronavirus disease 2019
CPAP	Continuous positive airway pressure
CT	Computed tomography
ICU	Intensive care unit
NF-κB	Nuclear factor-kappa B
PaO_2_/FiO_2_	Arterial oxygen partial pressure to inspired oxygen fraction
PEEP	Positive end-expiratory pressure
SpO_2_/FiO_2_	Oxygen saturation percentage to inspired oxygen fraction
SPSS	Statistical Package of Social Science
VCV	Volume-controlled ventilation
VV-ECMO	Veno-venous extracorporeal membrane oxygenation
